# Barometrical Pressure as a Factor in Medicine

**Published:** 1898-11

**Authors:** T. Wesley Burwood

**Affiliations:** L. K. Q. C. P. and L. M., Ireland, L. R. C. P. and L. M., Edin.


### BAROMETRICAL PRESSURE AS A FACTOR IN MEDICINE.

By T. Wesley Burwood, L. K. Q. C. P. and L. M., Ireland, L. R. C. P. and L. M., Edin.

Towards the end of the month of January, 1882, I was brought face to face with a puzzle which gave me much personal chagrin, though it eventually has proved a source of much satisfaction, as it opened up a line of thought which to me has been very useful.

How frequently in the experience of all of us have our failures been fruitful for good.

I had been in regular attendance on a gentleman whose name is well known. He had been my patient off and on for some years for trivial ailments, and I flattered myself I knew everything it was possible to know about him; he had been a most healthy man, never having had a serious illness since childhood. His habits were those of an English gentleman, and until a few months before his death he might have been seen on horseback in "The Row" every morning of his life when in London. He, however, towards the end showed signs of œdema in the feet due to heart trouble. There was no valvular lesion, though latterly marked dilatation was evident. There was no albumen in his urine and his present condition might be summed up as due to old age (eighty-four).

I had seen him one day about 6 P. M.; he was jolly and jocular, with nothing to indicate anything like a sudden collapse, his respiration was normal and his pulse and temperature satisfactory.

To my surprise, when I called about noon the next day I found my patient dead!

On inquiring of the nurse, who was a woman of experience, what had happened, she said: "He went to sleep as usual between 10 P. M. and 11, but woke soon after 1 complaining of breathlessness, his heart beating very fast and irregularly; this went on until gradually he became pulseless, and he breathed his last at 6 A. M., five or six hours after first awaking."

When I left him the previous afternoon I told the friends I considered his condition highly satisfactory, and yet within twelve hours he was dead!

I felt my position acutely, as I feared lest the friends might consider I had not fully grasped the situation, and that my reputation was endangered, which, however, did not prove to be the case.

During the rest of the day I turned over in my mind every possibility that could have arisen, but derived no satisfaction from any of my cogitations.

Taking up the evening paper after dinner I saw an account of a "Terrible Gale," which led me to wonder whether *that* had anything to do with my patient's sudden death. And this led me to the outcome of this paper.

Now I was not, nor am I, a meteorologist, but the facts are these: For four or five weeks before my patient died the weather had been what is commonly known now as "Anti-cyclonic," and on referring to the charts published daily by the Meteorological Society I found the barometer had stood between 30.2 and 30.8 for nearly five weeks. But about midnight there was a sudden fall, the glass dropped to 29.1, and before the gale was over it had fallen to 28.5.

Now what does all this mean? I am not going to give you a lecture on the barometer, we all know what that is, and few look at the instrument as it hangs in their entrance hall, excepting simply to see what the weather for the day is likely to be. To me it *is* that, but it is also a great deal more.

Now we all know when the barometer stands at 29 inches it means the atmospheric pressure registers 15 pounds on every square inch at sea level, consequently when the glass indicates 30 inches there must be more than 15 pounds pressure to the square inch, and more still as the Mercury is forced up to nearly 31 inches, which latter point I have seen only once in sixteen years' daily watching every morning and night.

Professor Darwin, in an article in the *Fortnightly Review*, says: "The barometer ranges through fully two inches. Hence, when the barometer is very high, every square foot of the earth's surface supports about 140 pounds more than when it is low, and 140 pounds to the square foot is 1,800,000 tons to the square mile."

What then happened on this sudden fall taking place? My explanation is—my patient's heart had been beating strenuously for some weeks against all this extra pressure, and when this pressure was suddenly taken off, like a horse going up hill with a heavy load behind him, the traces suddenly breaking the horse gallops away free—the heart's action was increased, the over-loaded heart and lungs became oppressed, a clot was gradually formed in the cardiac cavities, and soon life became extinct.

I have during all these years of observation seen very many cases of one kind or another so affected, and I can assert, without fear of contradiction, that any one interested in this subject will find notices of sudden death in the obituary of the morning papers after a sudden rapidly falling of the glass accompanied by a gale of wind or a hurricane; and many of these are cases, which, no doubt, have been a puzzle to the medical men in attendance, when their patients have suddenly passed away without any apparent cause.

You may have a powerful heart bounding and thumping away, driving its blood with increased violence into the cerebral vessels, and should there be a weak, atheromatous spot, cerebral apoplexy is the result, and your patient, who goes to bed apparently quite well, is found lifeless in the morning.

This applies also to epistaxis, pulmonary, and renal hemorrhages, angina pectoris, also in pruritus vulvæ, and many other conditions.

This brings me to an interesting point, which has frequently been discussed, though I have never seen it mentioned in connection with my subject—*that is*, why so many deaths take place in the night or early morning? I believe this is due to conditions to which the barometer can testify.

Captain Greenstreet, R. N. R., a man of great intelligence, who made observations extending over many years and in every part of the globe, showed me the automatic readings from his aneroid, and said "that it mattered not in what part of the world he was, there was always a slight and sometimes great falling in the Mercury between 3 and 5 A. M.," and my theory is, I think, substantiated thereby.

There is not a medical man of exprience present but can call to mind patients so susceptible and sensitive, that before they get out of bed in the morning they are able to tell that the wind is easterly, while others, who may not be affected by the east wind, will emphatically tell you there is going to be thunder, and you may rely on their prophecy coming true.

That there is in some patients what I call "meteorological susceptibility" I can prove, as numbers of my patients keep an aneroid in their bedrooms, which they watch night and morning, and so regulate their action and mode of living accordingly.

My friend, Dr. Reed Hill, when he was living with me was so aware of this fact that if he were disturbed in the night by a gale of wind he would say at breakfast, "We shall get a telegram directly from Mrs. So and So," and surely enough before noon that telegram came; or I would say, "We must look after Mr. So and So's heart while this gale continues," and we invariably found it necessary, for the patients had had restless nights, with wakefulness and palpitation which nothing could account for.

Many years ago I was attending an elderly lady suffering from bronchitis and weak heart. During the progress of her illness the barometer ranged very high, and on coming downstairs after visiting her one day, I was met by the patient's son-in-law, a retired judge, who inquired how Mrs. —— was?

I replied, "She is going on as well as she can, and as long as the barometer stands as high as it does there is no immediate danger, but if there should be a sudden fall we may expect diastrous results." In less than a week the Mercury fell one night, a gale sprang up, and on going to the house next morning I inquired of the footman how my patient was, and was told she had had a restless night. On reaching her bedroom I found the family watching her breathe her last.

That same morning between 5 and 6 o'clock I was called to see an old lady of eighty-four, who had had a bad attack of dyspnœa, palpitation, and diarrhœa in the night, and who died shortly before 9 o'clock the same morning.

That same afternoon at or about 3 o'clock the public was shocked with the news of the sudden death of Sir S. Northcote at the Foreign Office.

It may be interesting to the members if I recall to them the sudden death of Archbishop Benson in Hawarden Church, which took place during a hurricane and a rapidly falling barometer.

In the spring of 1887 there were several letters in the *Lancet* from different medical men, asking if any of their *confreres* could account for so many calls to patients suffering from diarrhœa, the attacks coming on on a certain Saturday. The same inquiry was repeated in the next week's issue. I may say, in passing, I looked carefully, and found no reply was ever sent.

On this same Saturday, when I went in to luncheon, I found a telegram from a patient I had recently taken to Brighton, and before the meal was over I had another from a patient in Essex, and a third in Acton, and a fourth in Hanwell, all of them with sudden attacks of diarrhœa. For the next few days I was busy with fresh cases, all of which dated their ailments from about midday on Saturday; some were men, some were women, all under different conditions as to health, locality, and age. Nothing in the shape of indiscretion in diet could account for it. For three weeks previously the weather had been anti-cyclonic, the barometer standing from 30.2 to 30.6. On the Friday evening the glass showed signs of downward movement, and by midday on Saturday a gale of wind had come into activity, and with it quickened action of circulation, more blood was driven through their susceptible livers, more bile thrown out, peristaltic movement increased, and in all these cases Merc-corrosivus quieted this internal disturbance and held the trouble in check.

Another instance in connection with this alteration in barometrical pressure occurred during a summer holiday in Switzerland. I was stopping with my wife and one of my daughters at Engleberg (3,314 feet above the sea level), and at the same hotel I found a well-known West End physician who arrived twenty-four hours before we did. We hobnobbed together and sat at the same table. On the third morning after his arrival he came to me after breakfast, asking me to prescribe for him for an attack of diarrhœa. On inquiry he said "they" had given him some bad salmon for dinner the day of his arrival, and this was the cause of upsetting his sensitive liver. Others who sat at the same table, and who had been in Engleberg some days and partook of the same were not affected. My friend said he had taken rhubarb pills to no purpose, so he asked me what he should do. I said "leave physic alone, eat as usual, and drink only Cognac until the bowels are quiet."

I asked him how long he had taken to get from Harley street, and whether he had rested *en route*. He said he had left Charing Cross at 11 A. M., and in the afternoon of the following day reached Engleberg. I suggested he should give the fish the benefit of the doubt, and that the diarrhœa was most likely due to his *rapidly* rising to the elevation in which he now found himself. I explained had he quietly stayed at Lucerne twenty-four hours, which is only 1,437 feet above the sea, and accustomed his heart to the altered pressure, it might have been altogether different.

Many visitors in Switzerland, to whom time is precious, rush away from London to find themselves in Alpine heights before they hardly know where they are, and consequently are frequently attacked by the "malade de montagne," which natives never experience.

Some years ago I had the widow of a clergyman under my care, who suffered from attacks of cardiac irregularity, palpitation, and dyspnœa. She had no cardiac lesion. There never seemed to be any cause, as far as she knew, for the attacks, which were very distressing to herself and alarming to her friends. On one occasion I was sent for, and found her with a tumultuous, irregularly throbbing pulse, and much distress. The attack came on in the early morning, and when I arrived about noon she was very exhausted, and looked it. Her temporal arteries were working synchronously with her radial pulse and her heart. During this time we were passing through an equinoctial gale. I at once gave her brandy, on the *similia similibus* principle, and gave strict injunction as soon as the breathing was easier and the heart's action quieter to stop the brandy, and moreover to watch the barometer and give small quantities of stimulants only on a falling glass. Some weeks after this, one tempestuous Sunday morning about 11 o'clock, I happened to be passing the house and casually dropped in to see how the atmospherical disturbance was affecting her. I found a messenger had been already sent for me, but I had left home before his arrival. I was met at the door by one of the daughters, with tears in her eyes and almost choked with her sobbing, saying, "I was only just in time." I hurried upstairs and found the bed surrounded by members of the family all weeping. The patient was in a state of loquacious delirium, saying she was "so happy," and with clasped hands, saying she was "seeing angels ascending and descending." I inquired how long this had been going on, and was told she had awakened about 5 A. M., very distressed as usual, and had been "rapidly sinking" ever since. Her pulse on my arrival was most regular and full, 110 to 120, and not the least like what I had noticed on previous occasions. I inquired how much brandy had been given, when a bottle of Martell's * * * was shown me, the contents of which, except a little at the bottom, had been given since she awoke. I told them there was no need for further stimulants for the next twenty-four hours, and at the end of that time she would be her usual self. Needless to say the angels all disappeared, and on my next visit the following day she had recovered from the intoxication, which her too fond children had helped her to induce.

The old lady has since died of senile decay, finally expiring during an equinoctial gale last spring.

Another condition which I have found almost invariably affected by the sudden lowering of atmospheric pressure is purpura hæmorrhagica. Mrs. ——, a lady nearly sixty years of age, has during the last five or six years been subject to purpura. She always knows when she is developing purpuric spots by the local pains, and these attacks are always more present during the period of a rapidly falling glass. On one occasion she suddenly became deaf in one year during a gale of wind, and when I saw her I diagnosed hemorrhage in the tympanum, which was confirmed by a West End aurist of great repute. In the summer of that year she took a house in an elevated position in the Lake district, and almost as soon as she arrived she suffered with palpitation of heart and fresh accession of spots. When she became accustomed to the elevation her cardiac action became regular, and the remaining part of the visit was happy and free from unpleasant symptoms, unless a gale of wind happened to arise.

Now in connection with ears, one often sees patients suffering from noises in the head, who will tell you that the degree of severity differs very much—some days very little, on others it is quite unbearable. If you suggest to them to watch the indication of the aneroid, they will tell you they are always better on a rising, and worse on a falling glass.

Another class of cases in which I have been very interested is epilepsy, and often have been astonished at the coincidence of epileptic attacks with rapid lowering of atmospheric pressure. In connection with this I was surprised on one occasion to find the father of a young lady under my care had made observations for the last ten years in connection with his daughter's attacks, and he found she was always well during a rising glass, or a prolonged anti-cyclonic period, but she always had an attack when the Mercury rapidly fell, and this usually in the early hours of the morning.

Another patient, a sweet, lovely little chappie of eleven years, is always more free from his attacks during days and weeks of anti-cyclonic periods, but recently he had nineteen fits in five days on a falling barometer.

On one occasion, in November, 1897, one Monday morning at 6 o'clock, four of my epileptic patients had attacks at the same hour, and this was eighteen hours after my pocket aneroid had registered 31 inches at the end of Hastings Pier, but at the time of their attack, the glass had fallen suddenly to 29.5.

I am not at all inferring that all cases of epilepsy are due to this cause, for we know they are not. Still, as so many epileptics do have their fits in the early morning, I think I am justified in saying in all probability they are induced by rapid alterations in atmospheric pressure affecting the circulation in the brain.

Another feature and interesting fact in connection with this subject. In the prolonged anti-cyclonic periods which sometimes prevail for weeks together, there may be at the same time an absence of rain, and consequently our drains and sewers are lacking water, while the atmospheric pressure keeps down and imprisons the sewer gases.

Some years ago I demonstrated this in connection with an epidemic of diphtheria. I was inquired of by the Medical Officer of Health whether I had among my patients any cases of diphtheria or sore throats. I replied, "I had no more under treatment than usual after a prolonged period of drought, whether that drought was caused by an absence of rain in summer or by frost in the winter."

In studying carefully the meteorological phase of the epidemic, I found the outbreak took place on January 22d. Five weeks previously, *i. e.*, from December 17th, a period of thirty-six days, there had been no rainfall at all, consequently the drains were in a state of quiescence. Between these same dates our average height of the barometer was 30.30; this showed the atmospherical pressure was of very high range and spread over a long period. Consequently, when the barometer falls, this great pressure being taken off, the obnoxious imprisoned sewer gases are liberated and escape through faulty joints and defective traps and valves. Given a long period with a high atmospheric pressure, coupled with defective closets and drains, one can predict, almost to a certainty, when the glass falls there will not only be sporadic cases of diphtheria and diphtheritic throats, and follicular tonsilitis, but in districts where numbers of houses have their closets, etc., faulty, there will be in all probability an epidemic of the disease.

If at the time of the fall there is a gale of wind to blow away the miasm all well and good, but if there is a little or no wind the gases are not easily dissipated. A falling barometer and a dead calm are very important factors, and in this case, on January 14th the barometer began to fall, and continued doing so steadily during the 15th, 16th, 17th, and 18th, which days I looked upon as the incubation period of the epidemic, as there was a dead calm on the 14th, very little wind on the 15th, and still less on the 16th and 17th.

From what I have advanced, I wish it to be distinctly understood I only find these conditions in the patient on a sudden and rapid falling of the glass. A northeast gale may be raging with fury, the glass rising all the time, and during its continuance the patient may be delightfully comfortable, but it is when the storm suddenly subsides and the Mercury runs down the patient is distressed.

On a slowly progressive downward tendency of the glass the patient is not so much affected, as he has had time to accommodate himself, though unwittingly, to the altered circumstances by which he has been surrounded.

Now with regard to treatment. This must be carried out, in my opinion, by each patient having the homœopathically selected remedy suited to his own individual case, as much care being taken in the diagnosis of the medicine as in the diagnosis of the disease. In the majority of the cases, at the time of the attack brandy or whisky or ether, in small doses, will be most beneficial.

The usual cardiac remedies, all of which are so well known to the members present, will, of course, be found useful.

In the intervals, general constitutional treatment will be necessary to so fortify the patient that he may be able to battle with the trouble to which his peculiar idiosyncrasy has made him liable.

I do not wish my medical friends to infer I consider all diagnoses are referable to alterations in atmospheric pressure. But I do say, where every other factor is carefully weighed and no satisfactory conclusion is arrived at, the probabilities are that the barometer will settle the difficulty, especially when the disturbance is functional rather than organic.

Professor Darwin, in the article before mentioned, which was published in February, 1887, says: "It is found that earthquakes are indubitably more apt to occur when there is a rapid variation of the pressure of the air, indicated by a rise or fall of the barometer, than in times of barometric quiescence, and the connection between barometric variations and earthquakes should make us reflect on the forces brought into play by the rise and fall of atmospheric pressure."

Now why should not the human subject be interfered with by these same influences?

Our very familiarity with these changes may easily blind us to the greatness of the forces which are so produced, and I am convinced that many present, if they will take the same trouble and interest in it that I have done, will be equally satisfied.

Though there may not be enough to enlist the interest of the Congress in what I have advanced, I have found it very useful from an ætiological point of view, as well as a help in diagnosis, prognosis, and treatment.—*Monthly Homœopathic Review, August 1st, 1898.*

